# A support vector machine based drought index for regional drought analysis

**DOI:** 10.1038/s41598-024-60616-3

**Published:** 2024-04-29

**Authors:** Mohammed A Alshahrani, Muhammad Laiq, Muhammad Noor-ul-Amin, Uzma Yasmeen, Muhammad Nabi

**Affiliations:** 1 Department of Mathematics, College of Sciences and Humanities, Prince Sattam Bin Abdulaziz University, 11942 Alkharj, Saudi Arabia; 2 Department of Statistics, COMSATS University Islamabad-Lahore Campus, Lahore, Pakistan; 3 Department of Mathematics and Statistics, Brock University, St. Catharines, Canada; 4 Khost Mechanics Institute, Khost, Afghanistan

**Keywords:** Drought index, Machine learning, Support vector machine, Drought analysis, Statistics, Climate sciences, Environmental sciences, Environmental social sciences

## Abstract

The increased global warming has increased the likelihood of recurrent drought hazards. Potential links between the frequency of extreme weather events and global warming have been suggested by earlier research. The spatial variability of meteorological factors over short distances can cause distortions in conclusions or limit the scope of drought analysis in a particular region when extreme values predominate. Therefore, it is challenging to make trustworthy judgments regarding the spatiotemporal characteristics of regional drought. This study aims to improve the quality and accuracy of regional drought characterization and the process of continuous monitoring. The new drought indicator presented in this study is called the Support Vector Machine based drought index (SVM-DI). It is created by adding different weights to an SVM-based X-bar chart that is displayed with regional precipitation aggregate data. The SVM-DI application site is located in Pakistan's northern area. Using the Pearson correlation coefficient for pairwise comparison, the study compares the SVM-DI and the Regional Standard Precipitation Index (RSPI). Interestingly, compared to RSPI, SVM-DI shows more pronounced regional characteristics in its correlations with other meteorological stations, with a significantly lower Coefficient of Variation. These results confirm that SVM-DI is a useful tool for regional drought analysis. The SVM-DI methodology offers a unique way to reduce the impact of extreme values and outliers when aggregating regional precipitation data.

## Introduction

A natural phenomenon that occurs all over the world is drought. In many regions of the world droughts are becoming increasingly frequent and severe as mentioned by^1^. Coles and Tawn ^2^ define drought as a decrease in the amount of water available over a specific period and area. Precipitation stands out as the most crucial climatological factor influencing both droughts and floods. The variability in precipitation can give rise to either of these natural hazards. Wilhite ^3^ analyzed precipitation and drought climatologist offers valuable insights for enhancing water management strategies, environmental protection, agricultural production, and socioeconomic development in specific regions. Drought stemming from insufficient precipitation in a particular area is both a disaster and a naturally occurring hazard. Hirabayashi et al. ^4^ highlights that understanding precipitation and drought patterns can significantly contribute to the effective management of water resources, environmental preservation, agricultural practices and socioeconomic progress. Paulo et al. ^5^ discussed the frequency, severity and duration of drought exhibit variations across diverse climatic zones. Cai et al. ^6^ recognized that drought is one of the most impactful climatic extremes affecting a larger population than any other type of natural disaster.

Mohamadi et al.^7^ discussed that drought indices play a crucial role as tools for monitoring and assessing different types of droughts, including: (a) meteorological drought, denoting a period with insufficient precipitation over a region; (b) hydrological drought, linked to insufficient surface and subsurface water during a specific timeframe; (c) agricultural drought, typically associated with reduced soil moisture leading to crop failure; and (d) socio-economic drought, which is linked to a time when water resource systems are unable to meet demand for water. However, the meanings of drought are always changing to take into account its effects on society and the ecosystem. Kaur et al.^8^ presented that multitude of indices utilizing different variables have been devised to detect and measure occurrences of drought. Included in these are the Surface Water Supply Index (SWSI), the Rainfall Anomaly Index (RAI), the Streamflow Drought Index (SDI), the Palmer Drought Severity Index (PDSI), the Reconnaissance Drought Index (RDI), the Standardized Precipitation Index (SPI), the Standardized Precipitation Evapotranspiration Index (SPEI), and the Standardized Runoff Index (SRI). McKee et al.^9^ introduce the SPI which stands out as the most commonly employed indicator for evaluating meteorological drought, characterized by a brief decline in precipitation leading to reduced water resources availability and ecosystem carrying capacity. Vicente-Serrano^10^ mentioned exclusive consideration of precipitation simplifies the calculation process as compared to more intricate indices, enabling the comparison of diverse drought conditions across various temporal and geographical dimensions. Capra and Scicolone^11^ discussed that SPI is particularly robust and practical as it allows assessment over diverse time spans, facilitating the exploration of various drought categories.

Barker et al.^12^ discussed SPI is a non-linear method relies on conventional statistical approaches for drought prediction introducing a considerable level of uncertainty. The application of machine learning (ML) algorithms has been employed for SPI estimation. Support Vector Regression (SVR) has been employed by Borji et al.^13^ to use support vector machines for regression tasks, demonstrating effectiveness in estimating drought. Liu et al.^14^ utilize a single-layer feedforward neural network for swift and efficient learning, successfully applied in modeling drought. Deo et al.^15^ adopts regression splines to capture non-linear relationships, proving effective in estimating drought. Rhee and Im^16^ leverage extremely randomized decision trees for precise and robust drought modeling. Nguyen et al.^17^ integrate fuzzy logic and neural networks for adaptive modeling, achieving success in drought estimation. Banadkooki et al.^18^ use interconnected nodes to emulate the human brain's learning process, applying it to drought modeling with promising outcomes. Elbeltagi et al.^19^ employe an ensemble of multiple decision trees to enhance accuracy and reduce overfitting, demonstrating efficacy in drought estimation. Kushwaha et al.^20^ discussed that the Super Vector Machine (SVM) is a novel machine-learning algorithm has been recognized as a reliable approach for addressing complex data related issues. Achirul Nanda^21^ discuss the implementation of SVM boasts essential features such as advanced validation, geometric explanation, and precise statistical tracking all achieved with a relatively low number of training data sets. Sihag et al.^22^ utilizes the kernel functions play a crucial role in SVM model serving as a valuable tool in optimizing the dataset for a more accurate classification method. The prediction of drought is imperative for understanding future drought intensity, enabling effective planning to mitigate the impact of drought conditions and climate changes. Sakaa et al.^23^ discussed various models have been developed to forecast drought in semi-arid regions. These machine learning models exhibit the ability to predict information accurately by utilizing the correct input variables. This research clearly reveals a research gap concerning the application of machine learning algorithms in semi-arid environments and drought predictions, in contrast to earlier studies. The results of this work effectively address and overcome a sizable gap in the field of machine learning models for agricultural and meteorological drought prediction as well as drought forecasting.

This study aims to establish a statistical framework that improves the regionality and representativeness of diverse scattered observations within a specific area. As a result, the research introduces a novel tool for monitoring regional drought, grounded in an unequal weighting scheme based on the X-bar chart and SVM regression. This study specifically does: (1) It creates a new way to combine precipitation data from different stations in the region, called the SVM-DI, (2) It calculates the Standardized Precipitation Index (SPI) for each station and compares it to the new SVM-DI for the entire region, (3) It checks how well the SVM-DI compares to the regional SPI by looking at their correlations with each other. In simple terms, the study tries to make a better system for understanding drought in a specific area. It creates a new index called SVM-DI, compares it to existing methods, and checks how accurate it is by comparing it to the RSPI.

## Methodology

In the proposed methodology for the SVM-based X-bar control chart the first step involves the comprehensive collection of relevant data pertaining to the targeted process variable. Support Vector Machines (SVM) are employed to train the model, requiring the definition of input features and corresponding target values based on historical process data. In the following subsections, the SVM and the statistical control chart for the mean have been discussed.

### Support vector machine

The Support Vector Machines (SVM) for drought prediction/classification involves adapting the SVM framework to the specifics of monthly precipitation data. The mathematical equations would be similar to the general SVM equations, but with considerations for the features and labels related to drought.

The linear equation of the hyperplane for drought prediction is:

1 $$w.X+b=0$$

where, *w* is the weights of the vector, X is the feature matrix and b is the bias.

The objective function for SVM optimization to maximize the margin while ensuring correct classification would include the term:

2 $$minimizw\left(\frac{1}{2}{\left|\left|w\right|\right|}^{2}\right)$$

Subject to the constraints:

$${x}_{i}\left(w.x+b\right)$$ for all $$i$$.

The input features for SVM include historical meteorological data. Using the monthly precipitation data, we split the data into train and test. The featured variable is considered as time and the target variables is precipitation. By using the errors of SVM we make the X-bar control charts and detect the out-of-control points. The X-bar control chart is explained in the next section.

### Shewhart X-bar control chart

In statistics, the Statistical Quality Control (SQC) presents the many of control charts for the surveillance of both industrial processes and environmental processes^24^. In 1924, Shewhart introduced the first control chart, and since then various chart types have evolved to monitor diverse processes. Shewhart control charts are acknowledged as memory-less, signifying their lack of consideration for past information. These charts are particularly adept at identifying significant process shifts, prompting numerous studies aimed at enhancing their efficacy.

The x-bar control chart has the following mathematical structure:

3 $$\mu =\frac{{x}_{1}+{x}_{2}+\dots +{x}_{n}}{n}$$

4 $${\sigma }_{\overline{x} }=\frac{\sigma }{\sqrt{n}}$$

The UCL, CL and LCL are shown in equations as:

5 $$UCL=\mu +3{\sigma }_{\overline{x} }$$

6 $$CL=\mu$$

7 $$UCL=\mu -3{\sigma }_{\overline{x} }$$

### Proposed drought index based on support vector machine

This section provides the description of support vector machine-based drought index SVM-DI. As labeled in Sect. 1, SVM has important role in drought monitoring. In SVM-DI, we predict the values of standard error using the SVM. Next, we use the standard error of SVM to create the X-bar control chart. To do this we find the Out-of-Control Point (OCP) using the X-bar control chart. Additionally, the weights for the aggregation of region data are integrated using the cumulative count of OCP (COCP). The incorporation of SVM in the X-bar control chart is pivotal for the overall effectiveness of SVM-DI. It introduces machine learning adaptability, enhances drought prediction, and contributes to the identification of OCPs. This integration enables SVM-DI to capture unique regional drought patterns with improved accuracy and reliability.

**Figure 1:**
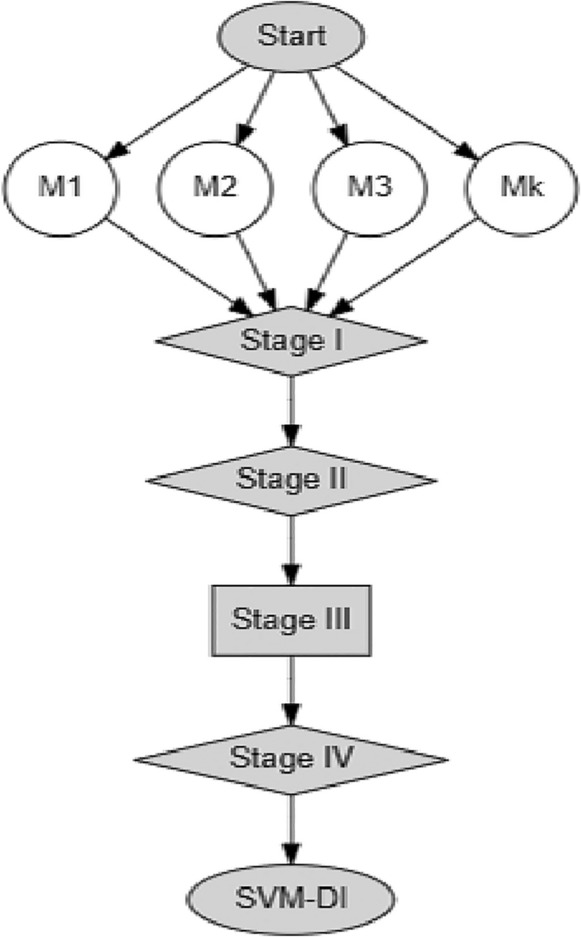
Flow chart of the SVM-DI.

Let $M\in {M}_{1}, {M}_{2}, {M}_{3},\dots , {M}_{k}$ represent the precipitation time series data from various meteorological stations within a designated area. The primary goal in this case is to create weights for the aggregation $M\in {M}_{1}, {M}_{2}, {M}_{3},\dots , {M}_{k}$ in a way that places stations with high COCPs at a lower weight than stations with low COCPs. Figure 1 shows the SVM-DI flow chart.

*Stage 1*: Incorporate of SVM based X-bar control chart.

We use X-bar control in the first stage of SVM-DI to detect the OCP. The UCL and LCL in the X-bar control chart are determined by averaging the time series data from all meteorological stations within a given geographic area.

Let ${Z}_{i}$ represent the meteorological station mean time series data. Under the X-bar control chart, we constructed the upper and lower control limits for ${Z}_{i}$. The vector with total values outside of the UCL and LCL is displayed by the ($COC{P}_{1},COC{P}_{2},COC{P}_{3},\dots , COC{P}_{k}$). Here, $i$ indicated the quantity of meteorological stations.

*Stage 2*: Weights Estimation.

In the second stage of SVM-DI, weights for the aggregation of time series data from multiple meteorological stations are determined. In this instance, the weight estimate is based on the COCP that is specific to each weather station. Assuring that observatories with relatively higher COCP are given lower weights and those with relatively smaller COCP are given higher weights are the following formulas.

8 $${v}_{i}=1-\frac{COC{P}_{i}}{U}$$

In the above equation, ${U}_{i}=\sum_{i=1}^{k}i.$

9 $${w}_{i}=\frac{{v}_{i}}{\sum_{i=1}^{k}{v}_{i}}$$

The condition is $\sum_{i=1}^{k}{w}_{i}=1$. Here, k denotes the number of meteorological stations.

*Stage 3*: Fusion.

After allocating the estimated weights for the regional precipitation data aggregation is the task of this stage in our process. Mathematically, the Weighted Mean Time Series Data (*WMTSD*) can be computed as follows:

10 $$WMTS{D}_{i}=\sum_{j=i}^{k}{M}_{ij}{w}_{ij}$$

$WMTS{D}_{i}$ denotes the regional precipitation data in the equation above using the suggested weighting scheme, where ${M}_{ij}$ denotes the time series data from the ${j}^{th}$ meteorological station and the ${w}_{ij}$ represents the estimated weights in the region.

*WMTSD* by considering the variability in the importance of meteorological stations through the COCP-based weights contributes to a more nuanced and accurate representation of regional drought. It plays a pivotal role in enhancing the reliability and effectiveness of SVM-DI as a drought monitoring tool. *WMTSD* is a critical component in this stage ensuring that the regional precipitation data is aggregated in a way that accounts for the significance of individual meteorological stations. Its role extends beyond aggregation, influencing the normalization process and ultimately contributing to the formulation of SVM-DI. This comprehensive approach enhances the ability of SVM-DI to accurately depict the spatiotemporal characteristics of regional drought conditions.

*Stage 4*: Normalization.

In SVM-DI, normalization is the final step. To model hydrological data, we have used KCGMD^25^. In this stage, the precipitation time series data are spatiotemporally aggregated regionally, and the Cumulative Distribution Function (CDF) of the KCGMD is fitted to them to normalize them. The KCGMD's CDF has the following mathematical expression:

11 $$F\left(q\right)=F\left(WMTS{D}_{i1}\right)+F(WMTS{D}_{i2}+\dots +F(WMTS{D}_{ik})$$

This paper derives SVM-DI using the normalization approximation proposed by Abramowitz and^26^. The normalization approximation's mathematical structure is provided by the equation that follows.

12 $$SVMDI=-(l+\frac{{d}_{o}+{d}_{1}l+{d}_{2}{l}^{2}}{1+{g}_{1}l+{g}_{2}{l}^{2}+{g}_{3}{l}^{3}})$$

$$l=\sqrt{ln (\frac{1}{{\left\{F\left(q\right)\right\}}^{2}})}$$

$$0\le F(q)\le 0.5$$

$$SVMDI=+(l+\frac{{d}_{o}+{d}_{1}l+{d}_{2}{l}^{2}}{1+{g}_{1}l+{g}_{2}{l}^{2}+{g}_{3}{l}^{3}})$$

$$l=\sqrt{ln (\frac{1}{{\left\{1-F\left(q\right)\right\}}^{2}})}$$

$$0.5\le F(q)\le 1$$

$${d}_{o}=2.515517, {d}_{1}=0.802853,{d}_{2}=0.010328, {g}_{1}=1.432788, {g}_{2}=0.985269, {g}_{3}=0.001308$$ are constant.

### The choice of K-component Gaussian mixture distribution

The computation of the SDI involves fitting a suitable probability distribution to the time series data of diverse climatic variables. The selection of an appropriate probability model demands careful consideration as the accuracy and reliability of estimates depend significantly on the fitness of the chosen model. Numerous univariate probability distributions have been proposed in previous research for modeling precipitation data in SDI calculations. The gamma distribution has been frequently employed by various authors in computing the SPI, as demonstrated by studies such as those conducted by^9^ and^27^.

In the last decade, researchers such as^28^ have put forth various probability models beyond the gamma distribution. The process of selecting the appropriate probability function can be facilitated through the utilization of R packages such as 'fitdistrplus' and 'Propagate,' as suggested by^29^. These tools contribute to the refinement of the SDI computation by aiding in the identification of a probability distribution that best fits the climatic variables' time series data, thereby enhancing the accuracy and reliability of the index estimates. The use of univariate probability models is deemed inadequate for achieving accurate inferences. Instead, employing a multi-model distribution or a mixture of probability functions is recommended to enhance computational accuracy. In line with this approach^30^, have proposed the utilization of K-CGMM (K-component Gaussian Mixture Model) for modeling precipitation time series. This advanced modeling technique considers a combination of probability functions, offering a more nuanced and robust representation of the underlying patterns in precipitation data. By adopting such an approach, the study aims to improve the precision and reliability of computational inferences related to precipitation, acknowledging the limitations of traditional univariate probability models in capturing the complexities of climatic variables.

## Application

**Figure 2:**
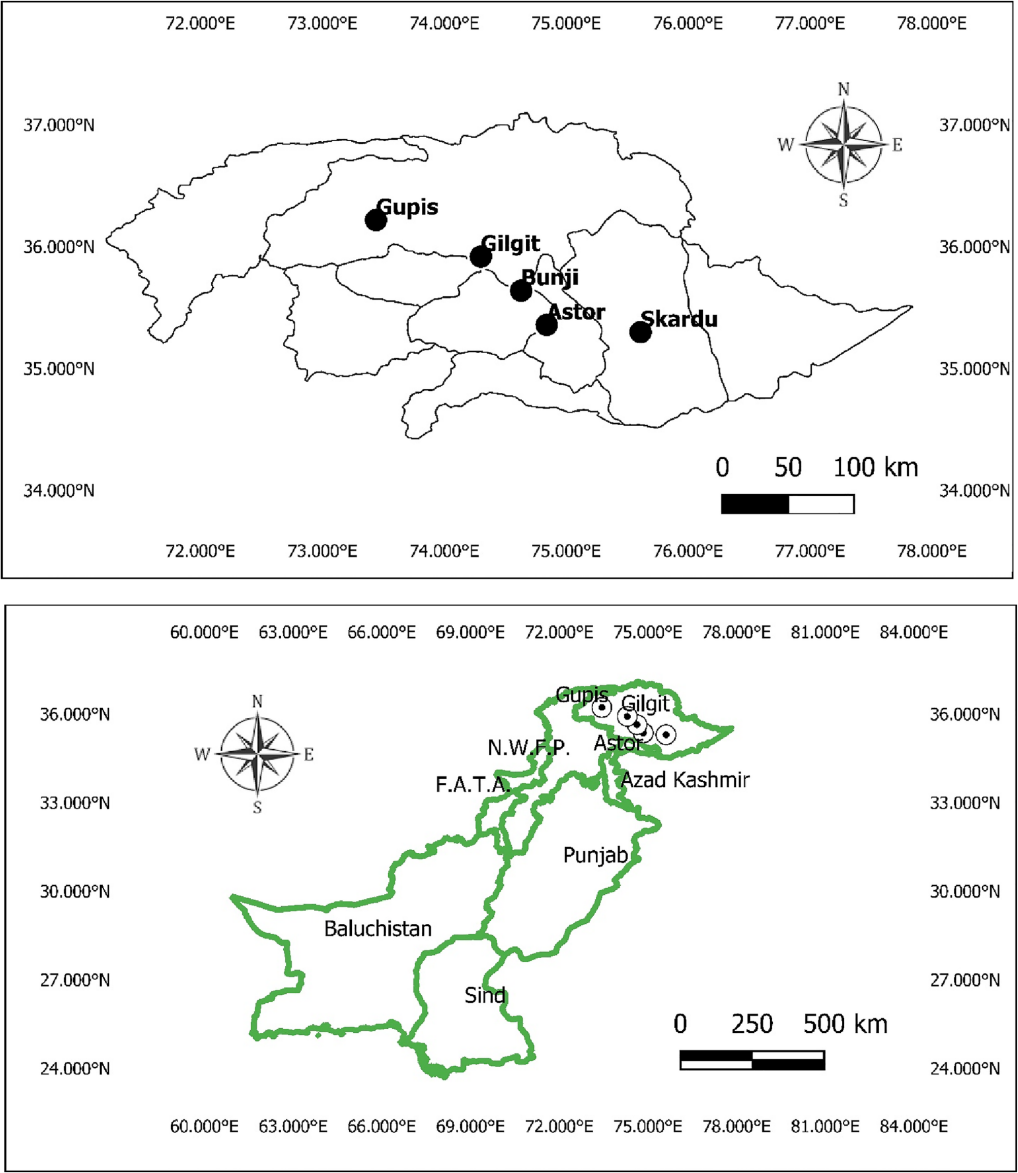
Meteorological stations chosen to assess the SVM-DI implications.

The central focus of the research application is the five weather stations located in northern region of Pakistan (see Fig. 2). This is situated on the second-highest plateau in the world, this selected region is of great significance to the nation's water resource management system. The dataset of selected meteorological stations consist of 41 years from January 1981 to December 2021, together with their corresponding latitude and longitude coordinates, are Astor (35.36 N, 74.84E), Bunji (35.64 N, 74.63E), Gilgit (35.92 N, 74.30E), Gupis (36.22 N, 73.44E), and Skardu (35.30 N, 75.61E). The challenges posed by climate change and global warming, the water resources in this region have experienced unprecedented depletion, leading to heightened risks of drought. This study evaluates the suggested model's implications for more precise regional drought monitoring by utilizing time-series data on monthly precipitation accumulation from 1981 to 2021. The source of the data is power data access viewer of NASA website. The arithmetic mean (AM), standard deviation (SD), and coefficient of variation (CV) in the correlation coefficient between the SPIs of distinct meteorological stations of the SVM-DI and RSPI. The Simple Mean Time Series Data (SMTSD) from all meteorological stations is normalized in this article to produce the RSPI. It's crucial to remember that the normalization used for the Weighted Mean Time Series Data (WMTSD) and SMTSD is the same. By following the^30^, the RSPI is computed by standardizing the simple average of time series (SATSD) data for all the stations. However, the standardization of SATSD is the same as that used for SVM-DI. The RSPI allows for the identification of wet and dry periods over different time scales, contributing to a nuanced understanding of the region's climatic variability. The RSPI is unable to handle the extreme values in the data where the SVM-DI is useful in this situation.

## Results and discussion

The proposed methodology is applied in this section. This section is further divided into three subsections.

### Detecting OCP using SVM based X-bar chart

This section presents and analyzes the outcomes concerning the identification of Out-of-Control Points (OCPs) by using residuals based X-bar control chart. The residuals are obtained by using the SVM based regression. The residual control charts are useful when the process exhibits autocorrelations^31^. The correlation in the Table 1 represents the correlation coefficient between the predicted values and the actual values of the target variable. The choice of the SVM based regression method is made on the basis of the correlation coefficient, mean absolute error, root mean square error, relative absolute error and root relative squared error results presented in the Table 1. Based on the results, SVM exhibited the most effective performance for precipitation (correlation = 0.6604, MAE = 0.5766, RMSE = 0.7280, RAE = 0.6853 and RRSE = 0.7524) when compared to the other models.

**Table 1:** Comparison of the machine learning models.

Model	Correlation	MAE	RMSE	RAE	RRSE
Support Vector Regression	0.6604	0.5766	0.7280	0.6853	0.7524
Decision Tree Method	0.5976	0.6631	0.8177	0.7819	0.8119
Gradient Boosting Method	0.6076	0.7106	0.8441	0.8279	0.8378

**Figure 3:**
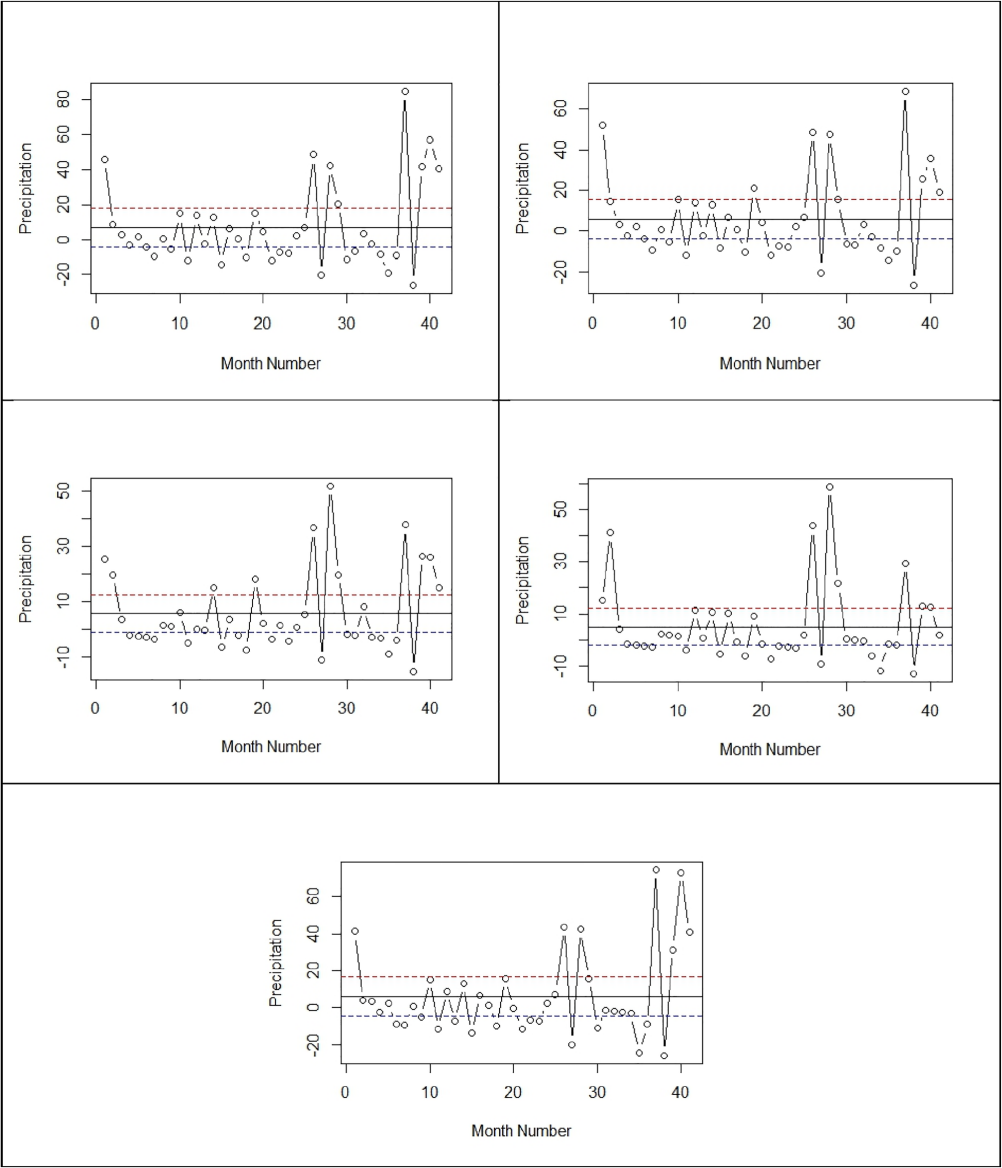
Control Charts Depicting OCP with UCL and LCL for each Station.

To improve the accuracy in achieving normality, we partitioned and organized the data on a monthly basis. For each month, UCLs and LCLs were estimated accordingly. The monthly data segregation and plotting for each station made it easier to identify OCP (see Fig. 3). The cumulative number of OCP recorded for each station during each month is displayed in Table 2. In January, 23 out of 41 observations from the Astor station were detected. In contrast, OCPs 23, 29, 21, and 22 have been found for Skardu, Gilgit, Gupis, and Bunji. Table 2 displays that the monthly distribution of OCPs in the precipitation time series data for each of the chosen meteorological stations.

**Table 2:** Monthly allocation pattern of weights and COCP.

Month	Astor	Bunji	Gilgit	Gupis	Skardu
January	23	23	29	21	22
Weights	0.2013	0.2013	0.1886	0.2205	0.2039
February	26	25	24	26	26
Weights	0.1988	0.2008	0.2027	0.1988	0.1988
March	32	32	29	24	32
Weights	0.1963	0.1963	0.2013	0.2097	0.1963
April	25	27	30	28	25
Weights	0.2037	0.2000	0.1944	0.1981	0.2037
May	32	32	29	24	32
Weights	0.1963	0.1963	0.2013	0.2097	0.1963
June	24	22	22	24	25
Weights	0.1987	0.2030	0.2030	0.1987	0.1966
July	27	26	30	22	26
Weights	0.1985	0.2004	0.1927	0.2080	0.2004
August	25	25	26	20	21
Weights	0.1966	0.1966	0.1944	0.2073	0.2051
September	19	20	26	24	22
Weights	0.2072	0.2049	0.1914	0.1959	0.2004
October	22	20	28	25	21
Weights	0.2026	0.2069	0.1896	0.1961	0.2047
November	24	21	25	24	21
Weights	0.1978	0.2043	0.1956	0.1978	0.2043
December	26	25	28	28	23
Weights	0.2000	0.2019	0.1961	0.1961	0.2058

### Weights estimation

These weights are estimated based on the degree of heterogeneity or homogeneity observed between stations in different months. The conclusions related to the weights derived from the recommended weighting scheme are discussed and clarified in this section. This method assigns weights to stations based on their COCP; stations with higher COCP are given higher weights, and stations with lower COCP are given lower weights. This makes sense because it is well-known that stations with lower COCPs are more closely aligned with the regional data, while those with higher COCPs diverge from it more. Therefore, it makes sense to assign higher weights to stations with lower COCP. Additionally, Table 2 displays the weight distribution for each station on a monthly basis under the suggested weighting scheme. These weights' spatiotemporal variation reflects how each station's significance varies from month to month.

### Consistency and efficiency of SVM-DI and RSPI

The Weighted Mean Time Series Data (WMTSD) log-likelihood and Bayesian Information Criterion (BIC) values for each time scale are shown in Table 3.

**Table 3:** Log likelihood and BIC for the KCGMD.

Time scale	Likelihood	BIC
1	698.3220	-345.0667
3	689.3926	-332.4133
6	8713.9890	-332.4248
9	732.7200	-329.5109
12	757.3073	-329.5215
24	842.1898	-322.8307
48	1034.0674	-320.5051

**Figure 4:**
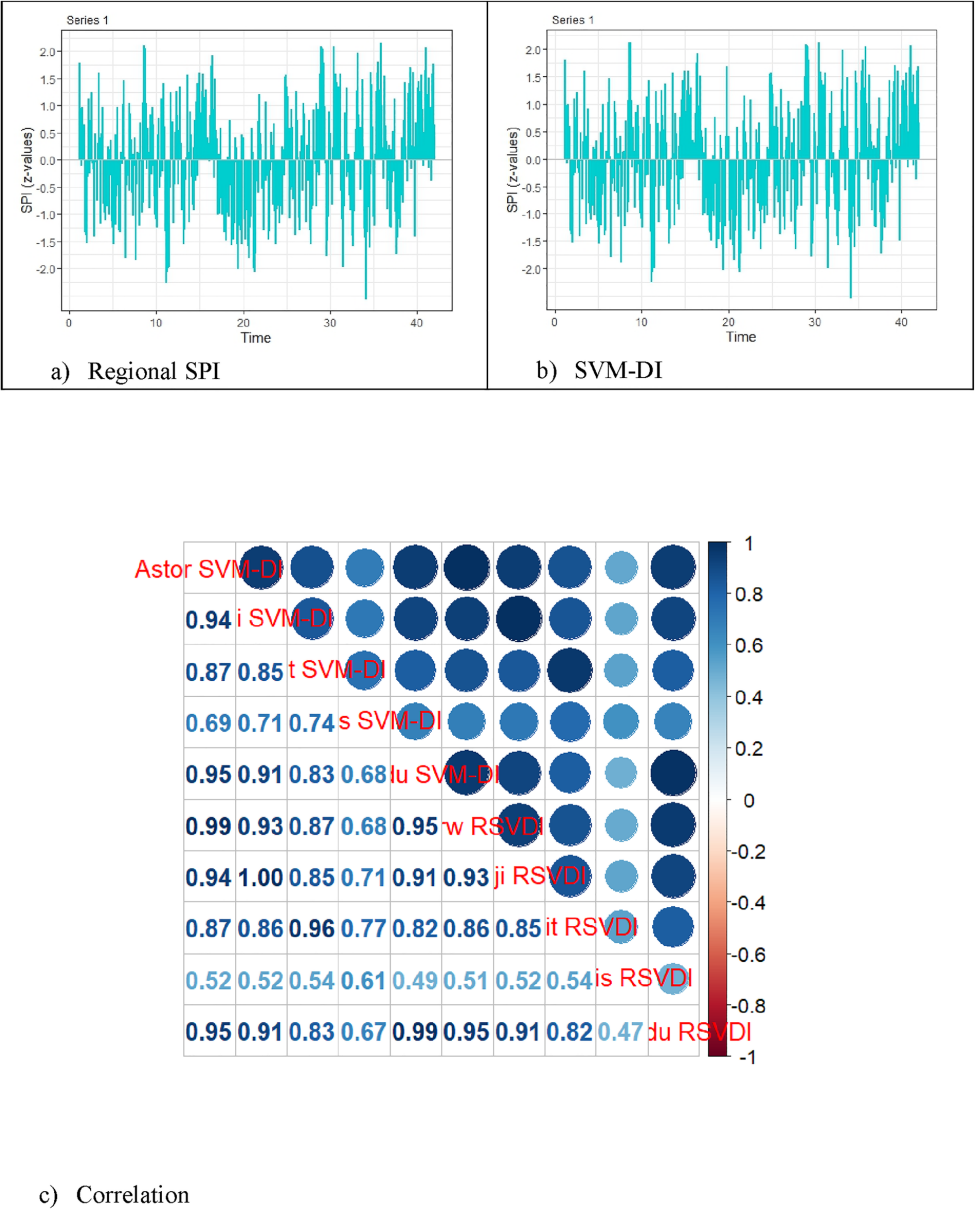
Correlation plot between SVM-DI and RSPI.

The log-likelihood values are significantly high, and the BIC values are consistently low on all time scales, confirming that the K-CGMD model is appropriate for the WMTSD data. Utilizing the Coefficient of Variation (CV) and Pearson correlation (r), one can evaluate the efficacy of the SVM-DI. We compared the SVM-DI and RSPI correlations with the SPIs of specific meteorological stations in order to assess consistency. The temporal and associative behavior of RSPI and SVM-DI are shown in Figs. 4a–c. Table 4 presents the correlations between the RSPI and SVM-DI and the data from specific meteorological stations-based SPIs over several important time intervals.

**Table 4:** Correlation analysis of SVM-DI and RSPI.

Time scale	Index	SPI				
		Astor	Bunji	Gilgit	Gupis	Skardu
1	SVM-DI	0.67081	0.66115	0.62813	0.62422	0.65529
	RSPI	0.67025	0.66054	0.60996	0.80401	0.64328
3	SVM-DI	0.72413	0.72931	0.70897	0.72974	0.74398
	RSPI	0.73191	0.72773	0.69119	0.82292	0.73922
6	SVM-DI	0.74952	0.75680	0.75636	0.77760	0.78271
	RSPI	0.76309	0.75499	0.75430	0.83331	0.77608
9	SVM-DI	0.76684	0.77382	0.77691	0.79308	0.80790
	RSPI	0.78293	0.77215	0.87449	0.84219	0.80484
12	SVM-DI	0.77394	0.75687	0.78381	0.79508	0.81655
	RSPI	0.78839	0.75499	0.79937	0.85501	0.81402
24	SVM-DI	0.81491	0.83387	0.83072	0.80651	0.86458
	RSPI	0.82529	0.83185	0.86979	0.89646	0.86543
48	SVM-DI	0.78151	0.85470	0.86477	0.81963	0.85631
	RSPI	0.78851	0.82525	0.89443	0.94726	0.85964

The Astor station has the highest correlation value of 0.67081 between SVM-DI and SPI on a one-month time scale, while the Gupis station has the lowest correlation value of 0.62422. Thus for Gupis and Skardu, the highest and lowest RSPI correlations with SPI are 0.80401 and 0.64328. For every other time scale, comparable ranges between the maximum and minimum correlation values are presented in Table 4. The discrepancies indicate that SVM-DI is more consistent than RSPI with regard to the SPIs of individual weather stations.

After the evaluation of consistency assessment, the effectiveness of SVM-DI in comparison to RSPI is assessed. The correlation coefficient statistics (mean, standard deviation, and coefficients of variation) for RSPI and SVM-DI are shown and contrasted in Table 5. The mean correlation between SVM-DI and individual meteorological stations is higher than RSPI at the one-month time scale. The observation that the standard deviation of individual meteorological stations is low implies that SVM-DI is more homogeneous than RSPI. Last but not least, SVM-DI is more consistent than RSPI, as indicated by the low CV in correlation values with the SPI of individual meteorological stations (see Table 5). All of these thorough results point to SVM-DI's greater regional emphasis than RSPI. This implies that the SVM-DI is a more appropriate tool for representing regional drought in an effective manner. The claim that SVM-DI is a more appropriate indicator for accurate and effective regional drought monitoring is firmly supported by provided results. In conclusion, water resource managers and policymakers will find the implications of SVM-DI to be beneficial, as they will provide deeper understanding of drought conditions and enable the creation of more efficient drought mitigation strategies.

**Table 5:** Assessment of Correlation Coefficients between SVM-DI and RSPI Values.

Time scale	Statistics	SVM-DI	RSPI
1	Mean	0.64790	0.67761
	Standard Deviation	0.02067	0.07429
	CV	3.18977	10.96396
3	Mean	0.72719	0.74259
	Standard Deviation	0.01260	0.04858
	CV	1.73313	6.54229
6	Mean	0.76459	0.77635
	Standard Deviation	0.01464	0.03303
	CV	1.91404	4.25395
9	Mean	0.78368	0.81532
	Standard Deviation	0.01661	0.04256
	CV	2.11926	5.22013
12	Mean	0.78519	0.80235
	Standard Deviation	0.02241	0.03659
	CV	2.85429	4.56061
24	Mean	0.83009	0.85776
	Standard Deviation	0.02226	0.02927
	CV	2.68185	3.41233
48	Mean	0.83538	0.86302
	Standard Deviation	0.03473	0.06138
	CV	4.15784	7.11288

## Conclusion

The impacts of global warming and climate change have resulted in frequent drought occurrences, significantly affecting various facets of life. To enhance regional drought monitoring, this paper introduces an innovative weighting scheme for the SVM-based X-bar control chart. As a result, the study introduces the SVM-DI, a novel regional drought index. The study focuses on several meteorological stations in Pakistan's northern region in order to assess the efficacy of SVM-DI. CVs in the correlations between RSPI and SVM-DI with specific meteorological stations are investigated for comparative analysis. The numerical results show that, in comparison to the straight forward RSPI, SVM-DI consistently displays more homogenous correlation values across all significant time scales. The higher mean correlation values for SVM-DI highlight its overall stronger association with meteorological stations' SPIs. The lower standard deviation indicates that SVM-DI performance is more stable and less susceptible to variations. These findings imply that RSPI is less able to adequately represent the entire region than SVM-DI. The study's findings demonstrate in summary that SVM-DI based drought monitoring is a useful method for examining drought characteristics at the local level. Practitioners of drought management can accurately define regional climatology with the help of these findings. This study is limited to the machine learning method by using precipitation data. One may enhance this study by incorporating some other covariates such as the temperature. The deep learning methods can also be utilized in multivariate case.

## Data Availability

All data analyzed during this study is obtained from the website of NASA power Data Access Viewers (1981–2021) and the website link is https://power.larc.nasa.gov/data-access-viewer/.
